# metaX: a flexible and comprehensive software for processing metabolomics data

**DOI:** 10.1186/s12859-017-1579-y

**Published:** 2017-03-21

**Authors:** Bo Wen, Zhanlong Mei, Chunwei Zeng, Siqi Liu

**Affiliations:** 1 BGI-Shenzhen, Shenzhen, 518083 China; 2 China National GeneBank-Shenzhen, BGI-Shenzhen, Shenzhen, Guangdong 518083 China

**Keywords:** Metabolomics, Pipeline, Workflow, Quality control, Normalization

## Abstract

**Background:**

Non-targeted metabolomics based on mass spectrometry enables high-throughput profiling of the metabolites in a biological sample. The large amount of data generated from mass spectrometry requires intensive computational processing for annotation of mass spectra and identification of metabolites. Computational analysis tools that are fully integrated with multiple functions and are easily operated by users who lack extensive knowledge in programing are needed in this research field.

**Results:**

We herein developed an R package, metaX, that is capable of end-to-end metabolomics data analysis through a set of interchangeable modules. Specifically, metaX provides several functions, such as peak picking and annotation, data quality assessment, missing value imputation, data normalization, univariate and multivariate statistics, power analysis and sample size estimation, receiver operating characteristic analysis, biomarker selection, pathway annotation, correlation network analysis, and metabolite identification. In addition, metaX offers a web-based interface (http://metax.genomics.cn) for data quality assessment and normalization method evaluation, and it generates an HTML-based report with a visualized interface. The metaX utilities were demonstrated with a published metabolomics dataset on a large scale. The software is available for operation as either a web-based graphical user interface (GUI) or in the form of command line functions. The package and the example reports are available at http://metax.genomics.cn/.

**Conclusions:**

The pipeline of metaX is platform-independent and is easy to use for analysis of metabolomics data generated from mass spectrometry.

**Electronic supplementary material:**

The online version of this article (doi:10.1186/s12859-017-1579-y) contains supplementary material, which is available to authorized users.

## Background

Biochemicals (metabolites) with low molecular masses are the ultimate products of biological metabolism, while a metabolome represents the total composite in a given biological system and reflects the interactions among an organism’s genome, gene expression status and the relevant micro-environment [1]. The most prevalent technology used in analysis of metabolomics is non-targeted mass spectrometry (MS) coupled with either liquid chromatography (LC-MS) or gas chromatography (GC-MS) [2, 3]. Generally, these techniques generate a set data of mass spectra with chromatography that includes retention time, peak intensity and chemical masses. Data analysis involves stepwise procedures including peak picking, quality control, data cleaning, preprocessing, univariate and multivariate statistical analysis and data visualization. A number of software packages are available for MS-based metabolomics data analysis as listed in Table 1, including propriety commercial, open-source, and online workflows. The MS manufacturers generally provide propriety software, like SIEVE (Thermo Scientific), MassHunter (Agilent Technologies) and Progenesis QI (Waters), which are often limited in scope and function. Open-source software, such as XCMS [4], CAMERA [5], MAIT [6], MetaboAnalyst [7] and Workflow4Metabolomics [8], usually cover limited processing steps. There is no such comprehensive pipeline that is used across the metabolomics community [9, 10]. Referring to the capabilities of the tools mainly used (as shown in Table 1), an automatic and comprehensive open source pipeline is urgent in bioinformatics analysis of metabolomics. Basically, the pipeline aims for users to easily perform end-to-end metabolomics data analysis with a flexible combination of different methods to efficiently integrate new modules and to build customized pipelines in multiple ways.

**Table 1:** Qualitative assessment of metaX compared to other existing metabolomics tools

No.	1	2	3	4	5	6	7	8	9	14	15	10	11	12	13	14
Feature	metaX	MAIT	Workflow4Metabolomics	MetMSLine	metaMS	MetaboNexus	MetaboAnalyst	XCMSOnline	MeltDB	Mzmine	Mzmatch	apLCMS	EigenMS	Metab	Metabomxtr	Metabolomics
Year	2015	2014	2014	2013	2013	2014	2009	2012	2008	2006	2011	2009	2014	2011	2014	2014
Language	R, Java	R	R, Perl, Python, Java	R	R	R	R, Java	R	perl, JavaScript and R	JAVA	JAVA, R	R	R	R	R	R
Platform independent	√	√	√	√	√	Windows only	√	√	√	√	√	√	√	√(windows & MacOS)	√	√
Open source	√	√	√	√	√	√	√	√	project- and user-specific access	√	√	√	√	√	√	√
Usable offline	√	√	√	√	√	√	√	-	-	√	√	√	√	√	√	√
Power analysis	√	-	-	-	-	-	√	-	-	-	-	-	-	-	-	-
Automatic outlier samples finding	√	-	√	√	-	-	-	-	-	-	-	-	-	-	-	-
PCA	√	√	√	√	-	√	√	√	√	√	-	-	-	-	-	√
Cluster analysis	√	√	√	√	-	√	√	-	√	√	-	-	-	-	-	√
PLS-DA	√	√	√	-	-	√	√	-	√	-	-	-	-	-	-	-
ROC analysis	√	-	-	-	-	√	√	-	-	-	-	-	-	-	-	-
Normalization	Sum, PQN, VSN, QC-RSC, ComBat, SVR, quantiles	-	Linear or local polynomial regression fitting	QC-LSC	-	Internal standard or quantile normalization	Normalized by sum/median, Normalized by reference sample/feature, sample specific normalization and quantile normalization	-	Normalized by specific compound or feature	Linear normalizaiton, normalized by internal standards	Normalized by Reference sample	-	combination of ANOVA and singular value decomposition	internal standard, medium, biomass(divides the intensity of each metabolite in a specific sample by the value of the biomass measured for this specific sample)	normalized using a mixture model with batch-specific thresholds and run order correction	normalized by sum,mean or media of each sample;normalized by specific reference;normalized by internal standards or optimal selection of multiple internal standards;
Biomarker analysis	√	-	-	-	-	√	√	-	-	-	-	-	-	-	-	-
Correlation network analysis	√	-	-	-	-	-	-	-	-	-	-	-	-	-	-	-
Metabolite identification	√	√	√	√	√	√	-	√	√	√	√	√	-	-	-	-
Functional analysis	√	-	-	-	-	√	√	-	√	-	-	-	-	-	-	-
Quality assessment	√	-	√	-	-	-	-	√	-	-	-	-	-	-	-	-
Peak picking	√	√	√	-	√	√	√	√	√	√	√	√	-	-	-	-
HTML-Based report	√	-	-	-	-	-	(PDF)	-	(PDF)	-	-	-	-	-	-	-

We herein developed a comprehensive workflow for analysis of metabolomics data, termed metaX. At the present time, R [11] is a popular statistical programming environment and provides a convenient environment for statistical analysis of metabolomic and other -omics data [12, 13]. We thus designed metaX as an R package that automates analysis of untargeted metabolomics data acquired from LC/MS or GC/MS and offers a user-friendly web-based interface for data quality assessment and normalization evaluation. This workflow, which is open source and rich in functions, encourages experienced programmers to improve the relevant functions or to build their own pipeline within the R framework. Overall, metaX aims to be a tool array that utilizes an end-to-end statistical analysis of metabolomics data.

## Implementation

**Fig. 1:**
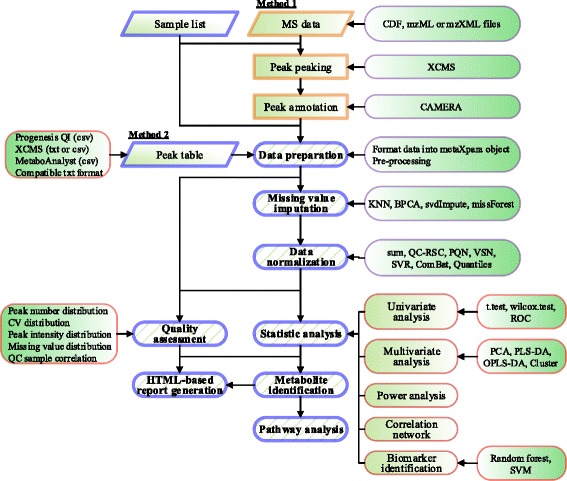
Overview of metaX. This figure summarizes the main modules, functions and features of metaX. The input data and the functions are included in the figure

A stepwise overview of data processing using metaX is illustrated in Fig. 1.

### Peak picking and inputs

In general, metaX can take mzXML files as input or a peak table file as input. If taking mzXML files as input, metaX will use the R package XCMS [4] to detect peaks, then use the CAMERA [5] package to perform peak annotation. If a peaks table file is an input, metaX transforms the table data from a peak detection software, such as Progenesis QI (exported comma separated value (csv) format file), into an R object compatible with the subsequent workflow.

### Pre-processing of raw peak data metabolite

The raw peak intensity data was pre-processed in metaX. Firstly, if a metabolite feature is detected in < 50% of quality control (QC) samples or detected in < 20% of experimental samples, it is removed from data analysis [14]. Secondly, a missing value after the first filtering is retained and imputed. In metaX, four methods are implemented to perform missing value imputation: k-nearest neighbor (KNN), Bayesian principal component analysis replacement (BPCA), svdImpute and random forest imputation (missForest) [15].

### Data scaling and transformation

Five different scaling approaches are offered in metaX: Pareto scaling, vast scaling, range scaling, autoscaling and level scaling [16]. The formulas of these scaling approaches are described in detail elsewhere [16]. In addition, three transformation approaches are offered in metaX: log, generalized logarithm (glog) and cube root transformation.

### Removal of outliers

metaX provides the ability to automatically remove the outlier samples in the pre-processed data based on expansion of the Hotelling’s T2 distribution ellipse [17]. A sample within the first and second component principal component analysis (PCA) score plot beyond the expanded ellipse is removed, and then the PCA model is recalculated. In default mode, three rounds of outlier removal are performed.

### Normalization

A metabolomics dataset usually contains unwanted variations introduced by signal drift/attenuation and multiplicative noise across the dynamic range. These effects can detrimentally impact the significant signal discovery and MS features that are required for rigorous quality assurance [14, 18]. In metaX, two types of normalization methods are provided: 1) Sample-based normalization is used to correct different concentrations of samples, such as normalization to total sum, probabilistic quotient normalization (PQN), variance stabilizing normalization (VSN) and quantile-based methods. 2) Peak-based normalization is implemented to correct data within batch experiment analytical variation and batch-to-batch variation in large-scale studies [19]. In this normalization, if a study contains QC samples, the QC-robust spline batch correction (QC-RSC) can be used to alleviate the effects of peak area attenuation [19]. During normalization, the degree of smoothening is controlled by a parameter that sets the proportion of points for smoothening at each point, while in metaX, this parameter is automatically assigned by using leave-one-out cross validation. On the basis of QC samples, a metabolite feature with a coefficient of variation (CV) over the predetermined value is excluded after normalization. The CV threshold could be set by users; generally, CV values ≤ 30% are recommended. Support vector regression (SVR) [20] and ComBat [21] normalization methods are also implemented in metaX. A user-friendly web-based interface (http://metax.genomics.cn) was offered for rapid evaluation of the data normalization methods for a specified dataset.

### Assessment of data quality

Pre- and post-normalization, the data quality is visually assessed in several aspects, 1) the peak number distribution, 2) the number of missing value distribution, 3) the boxplot of peak intensity, 4) the total peak intensity distribution, 5) the correlation heatmap of QC samples if available, 6) the metabolite m/z (or mass) distribution, 7) the plot of m/z versus retention time, and 8) the PCA score or loading plot of all samples. There are two ways to perform data quality assessment in metaX, the command line mode and the user-friendly web-based interface at http://metax.genomics.cn/.

### Univariate and multivariate statistical analysis

metaX offers both univariate and multivariate statistical analysis. For univariate statistical analysis, the parametric statistical test (Students t-test), non-parametric statistical test (Mann-Whitney U test), and classical univariate receiver operating characteristic (ROC) curve analysis are implemented. For multivariate statistical analysis, metaX offers functionalities for cluster analysis, multivariate modelling, including PCA, partial least squares-discriminant analysis (PLS-DA) and orthogonal partial least squares-discriminant analysis (OPLS-DA), with numerical and graphical results and diagnostics (optimal number of components estimated by cross-validation, R^2^, Q^2^, variable importance in projection (VIP), statistical significance of the model by permutation testing) [22]. In terms of the univariate test analysis, metaX also offers the false discovery rate (FDR)-corrected *p*-value by using the Benjamini-Hochberg FDR algorithm [23]. The PLS-DA was implemented based on the functions from the pls package [24], and the OPLS-DA was performed using the functions from the ropls package [25].

### Power and sample size analysis

metaX offers an easy-to-use function to perform the power and sample size analysis. This function is based on the Bioconductor package SSPA [26] and outputs a figure to show the distribution curve of sample size versus the estimated power.

### Metabolite correlation network analysis

metaX offers two types of network analysis. One is the correlation network analysis without regard for experimental groups information, and the other is differential correlation network analysis, which aims to identify metabolite correlation differences in a physiological state. The former was implemented using the cor function from the stats package to calculate the correlation coefficient, and the latter was implemented using the function comp.2.cc.fdr from the DiffCorr package [27] to calculate the significantly differential correlations. The igraph package [28] was used for network analysis and visualization. In addition, the network can be exported as a file in formats such as gml and pajek, which can be imported into Cytoscape [29] and Gephi [30] for network analysis and visualization. Both of the correlation network analyses aim to describe the correlation patterns among metabolites across samples, in which nodes represent metabolites and edges represent the correlation between different metabolites. The network analysis offers a complementary method to univariate and multivariate statistical analysis methods.

### Metabolite identification

Currently, metaX provides a function for metabolite identification based on the Human Metabolome Database (HMDB) [31], KEGG [32, 33], MassBank [34], PubChem [35], LIPID MAPS [36], MetaCyc [37] and PlantCyc (www.plantcyc.org). Moreover, metaX can easily be extended to support the other databases. The metabolites having molecular weights within a specified tolerance to the query m/z or molecular weight value are retrieved from the databases as putative identifications. The information of adducts and isotopes is utilized to assist in metabolite identification if it is present. The default tolerance is 10 ppm.

### Functional analysis

At present, metaX provides a function for metabolite pathway analysis based on IMPaLA [38].

### Biomarker analysis

metaX uses functions from the R package “caret” to perform the biomarker selection, model creation and performance evaluation [39]. Currently, two methods, random forest [40] and support vector machine (SVM), are implemented to automatically select the metabolites which show the best performance. After the best set features are selected, a randomForest model can be created and the ROC curve can be plotted.

### HTML-based report generation

metaX outputs an HTML-based report by using the Nozzle package [41], which contains quality assessment plots and other analysis results.

## Results and discussion

To illustrate the applications of metaX, a published non-targeted LC-MS metabolomics dataset from a coronary heart disease (CHD) study was used [42, 43]. The dataset consisted of two batches of 138 plasma samples (59 CHD patients, 43 healthy controls and 36 QC samples) acquired in positive ion mode on an LTQ Orbitrap Velos instrument (Thermo Fisher Scientific, MA, USA). LC-MS raw data files were converted to mzXML format using ProteoWizard (version 3.0.5941) [44] and then were processed by XCMS [4] and CAMERA [5] for peak picking and peak annotation, respectively. In total, 1438 features were retained for downstream analysis. The mzXML files can be downloaded from the Dryad Digital Repository [43]. It merits to note that the study focus is mainly on the software application and its capabilities, not on the biological interpretation of the generated results.

### Quality assessment of metabolomics data using metaX

**Fig. 2:**
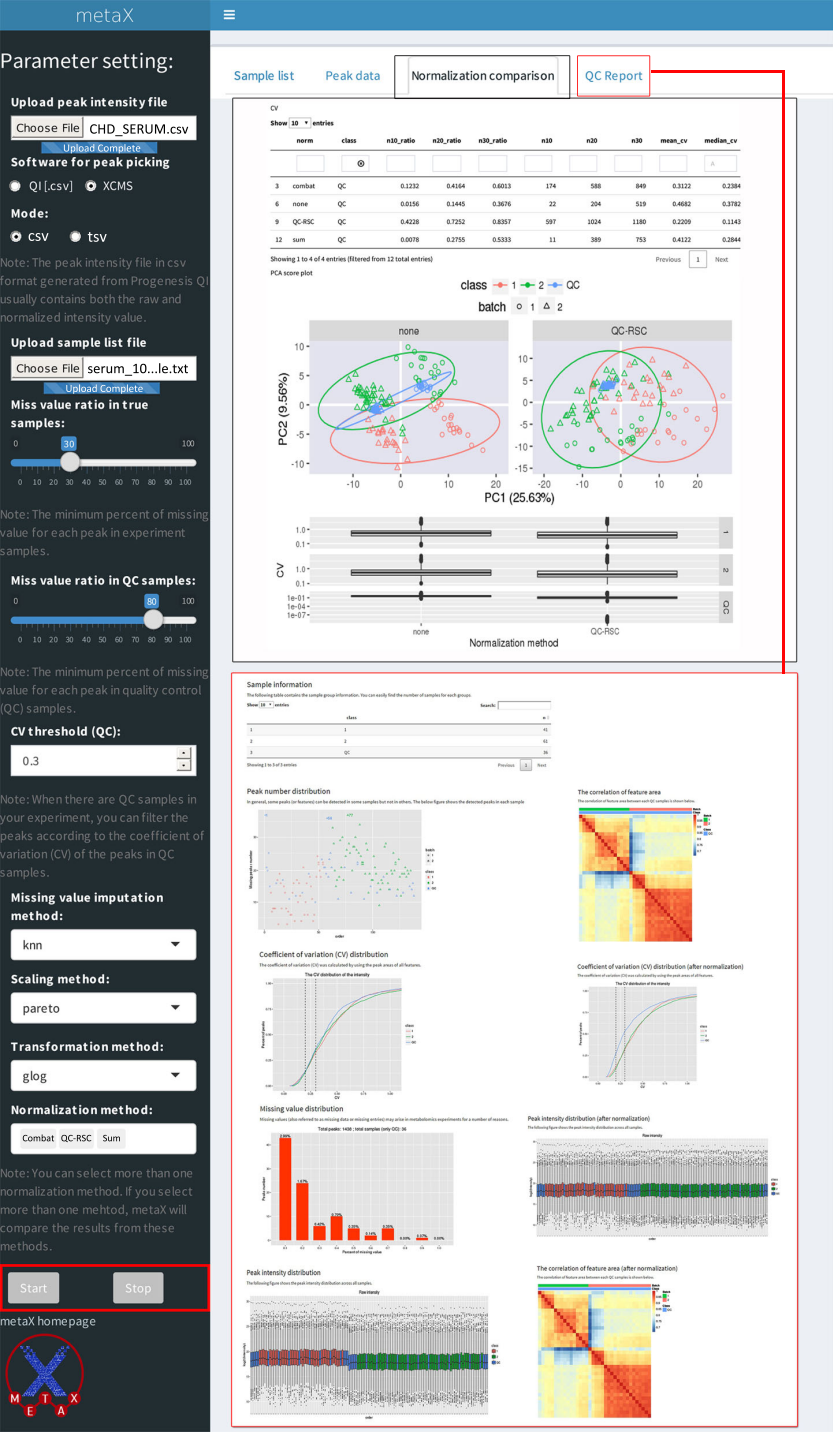
User interface of metaX for quality assessment and normalization evaluation

**Fig. 3:**
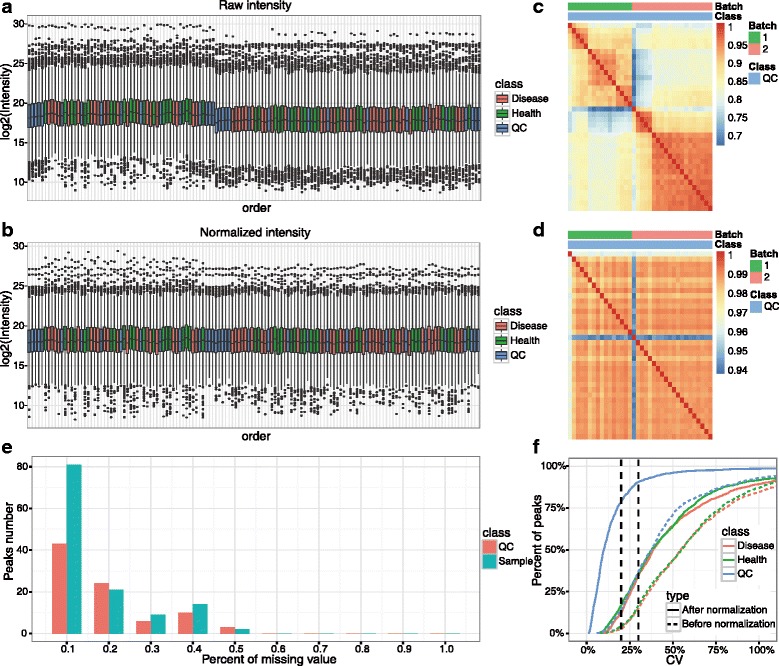
QC charts generated by metaX. **a** The intensity of feature distribution before normalization. **b** The intensity of feature distribution after normalization. **c** The correlation plot of QC samples before normalization. **d** The correlation plot of QC samples after normalization. **e** The missing value distribution in experimental and QC samples. **f** The CV distribution of all features before and after normalization for each group

**Fig. 4:**
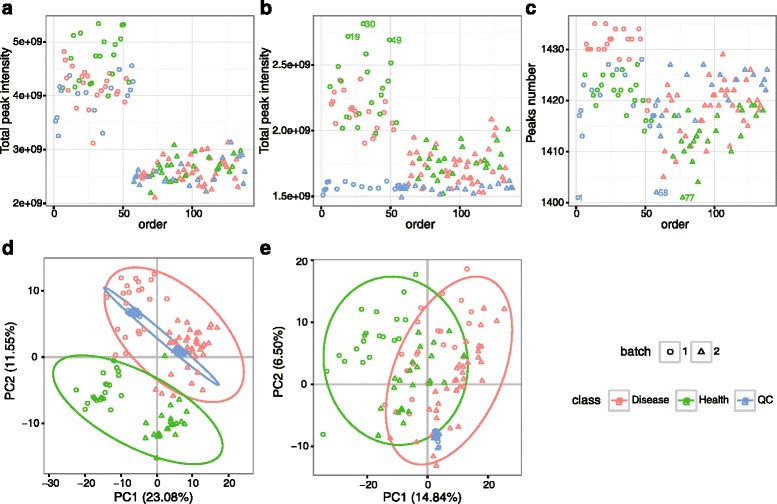
QC charts generated by metaX. **a** The sum intensity of all features per sample before normalization over the analysis time (injection order). **b** The sum intensity of all features per sample after normalization over the analysis time (injection order). **c** The number of features per sample over the analysis time (injection order). **d** The score plot of PCA for the raw feature intensity data. **e** The score plot of PCA for the normalized data

In metabolomics studies, data quality checks are crucial prerequisites to achieve reliable results. metaX offers a quick and easy data quality check of metabolomics data. This can be done using the R function in metaX or a user-friendly web interface at the website http://metax.genomics.cn/ as shown in Fig. 2. The mainly QC charts generated by metaX for the CHD dataset are illustrated in Figs. 3 and 4. The number of features detected per sample over the analysis time (injection order) is illustrated in Fig. 4c, revealing that the peaks acquired from any group, disease, healthy and QC, are randomly distributed. The intensities of all features per samples before and after normalization over the analysis time (injection order) are illustrated in Fig. 3a and b, respectively. The missing value distribution is shown in Fig. 3e, which gives an overview of the percent of missing values of all features in both the QC and experiment samples. According to Chawade’s view, the total missing value plot and the total intensity plot derived from raw data and treated with/without normalization could be used to identify sample outliers [45]. Our analysis supported this. The correlation plots of QC samples before and after normalization by SVR are illustrated in Fig. 3c and d and indicate that the lowest correlation efficiency is enhanced from approximately 0.7 to 0.9. The CV distribution of all features before and after normalization for each group is displayed in Fig. 3f, implying that after normalization, the signal quality is obviously improved. The sum intensity of all features per sample before and after normalization over the analysis time (injection order) is illustrated in Fig. 4a and b, suggesting that normalization could narrow the signal variation. The score plots of PCA for the raw feature intensity data and the normalized data are shown in Fig. 4d and e, respectively, which give an overview of the dataset and showing trends, groupings and outliers before data normalization and after data normalization. The score plot of PCA (Fig. 4d) for the non-normalized data provided a simple and easily interpretable visual check of the presence of batch effects. In Fig. 4d, the two data batches appear as two separated groups upon PCA analysis without normalization, whereas in Fig. 4e, after normalization the batch effect was reduced and all of the QC samples were clustered tightly, which provides an initial evaluation of the data quality. Overall, these QC charts demonstrate the necessity of normalization for metabolomics data, while metaX enables overview of the data quality with different charts.

### Evaluation of normalization methods using metaX

**Fig. 5:**
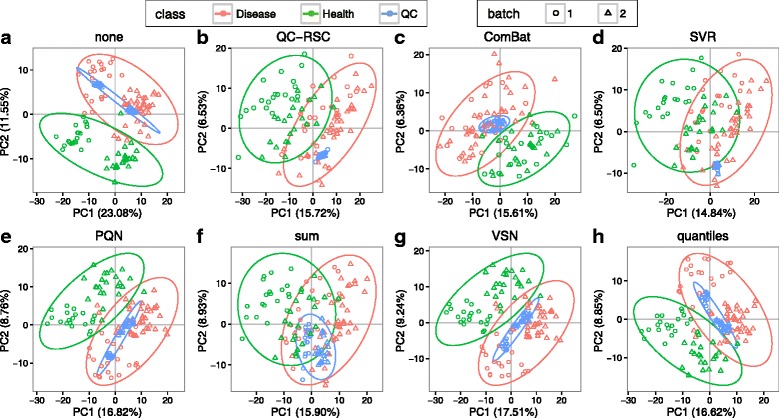
Comparison of different normalization methods from PCA. **a** none, **b** QC-RSC, **c** ComBat, **d** SRV, **e**) PQN, **f** sum, **g** VSN and **h** quantiles. The different points in the figures refer to different samples, and the samples were color-coded according to their group information and shape-coded according to their batch information

A systematic bias in high-throughput metabolomics data is often introduced by various steps of sample processing and data generation. Data normalization can reduce systematic biases. A question related to this issue is how to select a proper normalization method. metaX provides a user-friendly web-based Shiny application (http://metax.genomics.cn) for this purpose. To select the optimal normalization approach for the CHD dataset, seven methods are evaluated using metaX. Figure 5 shows the score plots of PCA using different normalization methods. They indicate that after normalization using QC-RSC, ComBat or SVR, all of the QC samples are clustered more tightly, and the batch effect is effectively reduced compared with other methods. Table 2 presents the quantitative comparison metrics acquired by the different methods. From the results it is clear that all normalization methods performed better than non-normalization used in most of the metrics. Specifically, SVR detects the largest number of features (1293) with CV ≤ 30% in QC samples, followed by QC-RSC (1191). For the average CV of features in QC samples, SVR achieved the best performance, followed by QC-RSC. This is similar to the findings in a previous study [20]. However, QC-RSC could detect the largest number of differentially expressed features (178), followed by SVR (170). Taken together, for this data set, SVR could be an optimal normalization method, thus it was chosen as the default normalization method for the downstream analysis.

**Table 2:** The comparison of different normalization methods

Methods	NO. of peaks	NO. of peaks (CV ≤ 30%)^a^	DEF^b^	Mean (CV) ^CHD d^	Mean (CV) ^Health d^	Mean (CV) ^QC e^
ComBat	1438	930	127	0.4261	0.3816	0.1636
none	1438	527	65	0.4865	0.4739	0.2114
QC_RSC	1438	1191	178	0.5108	0.4664	0.1098
SVR	1438	1293	170	0.4853	0.4583	0.1081
PQN	1438	793	125	0.4945	0.4681	0.1777
Quantiles	1438	740	118	0.4911	0.4646	0.1895
sum	1438	761	119	0.5044	0.4733	0.1979
VSN	1438	772	120	0.5014	0.4761	0.1912

*Note*:

^a^After normalization, the number of peaks with CV ≤ 30% in QC samples

^b^DEF: differentially expressed features with *q*-value < = 0.05, fold change > = 1.5 or fold change < = 0.667 and VIP > = 1

^c^Mean (CV) ^CHD^: The average CV of peaks in CHD disease group

^d^Mean (CV) ^Health^: The average CV of peaks in health group

^e^Mean (CV) ^QC^: The average CV of peaks in QC group

### Univariate and multivariate statistical analysis

Data for the QC samples are removed from the dataset prior to univariate and multivariate analysis in metaX. For univariate analysis, Mann-Whitney U test and Students t-test are performed to compare disease and health groups, followed by false discovery correction using the Benjamini-Hochberg method using metaX. The results, along with the fold change of the disease group versus health group, are presented in Additional file 1: Table S1. In total, 171 features (13.22% of total features) are detected under the criteria of the corrected *p*-value (Mann-Whitney U test) ≤ 0.05, fold change ≥ 1.5 or ≤ 0.667 and VIP > =1, and 170 features (13.15% of total features) are detected under the criterion of the corrected *p*-value (Students t-test) ≤ 0.05, fold change ≥ 1.5 or ≤ 0.667 and VIP > = 1. The result is comparable with that of the previous study [42].

**Fig. 6:**
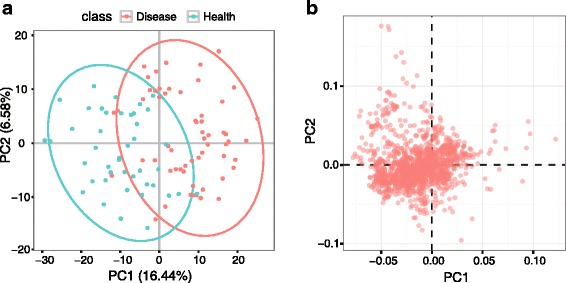
The score and loading plots of PCA. **a** Score plot of PCA and (**b**) Loading plot of PCA. The different points in the figures refer to different samples, and the samples are color-coded according to their group information. The QC samples were removed before performing the PCA analysis

**Fig. 7:**
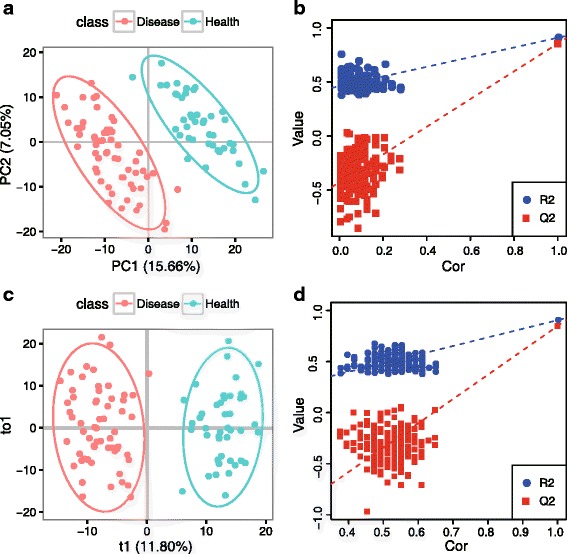
The score and permutation test plots of PLS-DA and OPLS-DA. **a** Score plot of PLS-DA. R^2^Y: 0.908, Q^2^Y: 0.854. **b** Permutation test plot of PLS-DA, *p*-value < = 0.05. **c** Score plot of OPLS-DA. R^2^Y: 0.905, Q^2^Y: 0.847. **d** Permutation test plot of OPLS-DA, *p*-value < = 0.05. The different points in the score plots (A and C) refer to different samples, and the samples are color-coded according to their group information. The number of permutations for the permutation test is 200

For multivariate analysis, PCA, PLS-DA and OPLS-DA are performed by metaX. In PCA analysis, the normalized peak intensity matrix is glog transformed, followed by Pareto scaling and centering, and then two components are selected. The PCA score and loading plots are shown in Fig. 6a and b, respectively. The score plot indicates that there is an apparent difference between the disease and health groups. For PLS-DA and OPLS-DA, the normalized peak intensity matrix is also glog transformed, followed by Pareto scaling and centering. Two components are selected for PLS-DA and two components (one orthogonal and one predictive) for OPLS-DA. The score and loading plots for PLS-DA and OPLS-DA are shown in Fig. 7a and c, respectively. The R^2^Y and Q^2^Y values of the PLS-DA model, which are 0.908 and 0.854, respectively, indicate that the model has good goodness of fit and predictive ability. The R^2^Y and Q^2^Y values of the OPLS-DA model, which are 0.905 and 0.847, respectively, indicate that the model also has good goodness of fit and predictive ability. Overall, the two multivariate data analysis methods, PLS-DA and OPLS-DA, give similar results. To test the validity of the models of PLS-DA and OPLS-DA, a permutation test (*n* = 200) is performed. As shown in Fig. 7b and d, the test indicated that the two models are valid, and the good predictive ability of the model is not because of over-fitting with a *p*-value less than 0.05. Taken together, the results of PCA and PLS-DA (or OPLS-DA) show a distinct separation between the disease and health groups.

### Biomarker analysis, metabolite identification and pathway analysis

**Fig. 8:**
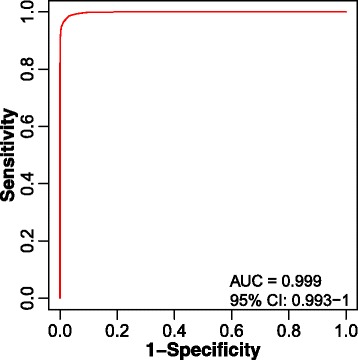
The ROC curve result of the six selected metabolites

To create the classification model between the disease and health groups, the functions implemented in metaX are used to conduct the biomarker selection, model creation and performance evaluation. A recursive feature elimination algorithm with the random forest model is used to select the best feature set. During the treatment, 5-fold cross-validation is used to optimize the model and reduce over-fitting. As shown in Table 3, 8 features were selected. To further evaluate the performance of the 8 selected features, the 102 samples were randomly split into two sample sets. One sample set (Disease: 29, Health 29) was for model building and the other (Disease: 14, Health 30) was for testing. Based on the two data sets, the 8 features were used to build a random forest model, and a receiver operating characteristic (ROC) curve of this model was plotted and is shown in Fig. 8. The result indicated that the model based on the 8 features had a good result with an area under the ROC (AUROC) curve of 0.999. The 8 features were then identified based on the HMDB (version 3.6) database through metaX. Seven out of the 8 features were identified with a mass accuracy of < 10 ppm (parts per million). The putative identified metabolites were then submitted to the IMPaLA website (version 9) through metaX to perform the pathway analysis, and the results are presented in Additional file 2: Table S2.

**Table 3:** The biomarkers selected by metaX

MZ	RT (min)	Mass	HMDB	Name	Delta (ppm)	Chemical formula
308.0498	10.46	285.0629	HMDB14387	Cladribine	−8.18	C10H12ClN5O3
424.3412	11.94	423.3349	HMDB06469	Linoleyl carnitine	−2.31	C25H45NO4
155.0281	2.81	116.066	HMDB32411	2-Methyl-1-methylthio-2-butene	−8.77	C6H12S
130.0499	3.43	129.0426	HMDB00267	Pyroglutamic acid	0.15	C5H7NO3
174.9913	2.30	NULL	NULL	NULL	NULL	NULL
309.0533	10.47	270.0892	HMDB33940	Vignafuran	3.44	C16H14O4
425.3446	11.94	424.3341	HMDB06327	Alpha-Tocotrienol	7.62	C29H44O2
324.0443	9.33	301.0563	HMDB01062	N-Acetyl-D-Glucosamine 6-Phosphate	−3.86	C8H16NO9P

### Correlation network analysis

**Fig. 9:**
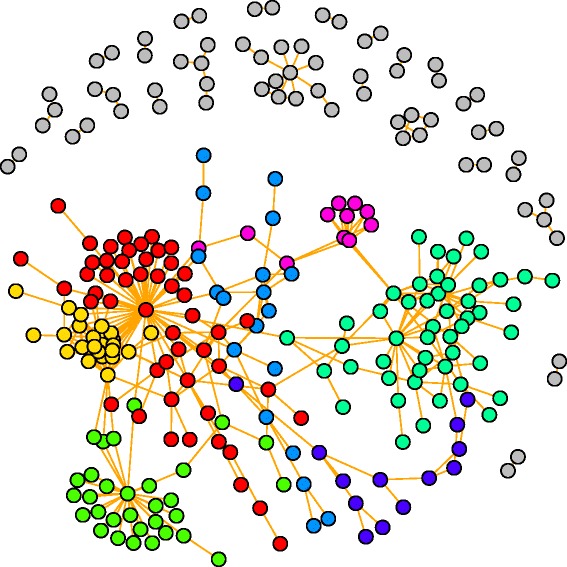
The differential correction network. The top six largest numbers of nodes communities were color-coded. Detailed information about the samples and their communities are presented in Table S3

Network-based correlation analysis is a complementary method to the traditional univariate and multivariate statistics that is taken in metabolomics analysis to identify metabolite changes in response to variable status of physiology. All of the features with the normalized intensity described above were used to perform the differential correction network analysis. This analysis can be used to detect the interconnection of metabolite pairs whose relationships are significantly altered due to the disease process. In this study, only the metabolite pairs that had significant differential correlations (*q*-value < = 0.01) between the disease and health populations were used to build the network. As shown in Fig. 9, of the network with 266 nodes and 444 edges, a giant component (198/266, 74.44%) was found and the community detection analysis using the fast greedy modularity optimization algorithm against this component resulted in seven communities, in which each one has equal to or greater than 10 nodes detected. In addition, metaX can estimate three centrality metrics (degree, closeness and betweenness) for each node, and they reflect the importance of the node in the entire network (Additional file 3: Table S3). Differentially correlation network analysis is expected to provide useful insights into the underlying biological processes of the clinical development of CHD.

## Conclusions

metaX presents a complete data processing software that is easy to operate and capable of dealing with large-scale metabolomics datasets. A metaX user can customize the pipeline according to the research requirements. Compared to software for metabolomics datasets that requires high-manual interaction, metaX requires much less manual interaction and can be used in a command line or web-based user-friendly interface. Based upon the fast process and the optimized workflow, therefore, metaX would greatly improve the interpretation of metabolomics data.

## Additional files

Additional file 1: Table S1. The fold change and *p*-value for all of the features. (XLSX 146 kb)

Additional file 2: Table S2. The pathway analysis results for the 8 selected biomarkers. (XLSX 14 kb)

Additional file 3: Table S3. The centrality metrics for each node in the network. (XLSX 22 kb)

## Abbreviations

FDR: False discovery rate
GUI: Graphical user interface
HMDB: Human metabolome database
MS/MS: Tandem mass spectrometry
OPLS-DA: Orthogonal partial least squares discriminant analysis
PCA: Principal component analysis
PLS-DA: Partial least square discriminant analysis
QC: Quality control
ROC: Receiver operating characteristic
